# Nutrition, One-Carbon Metabolism and Neural Tube Defects: A Review

**DOI:** 10.3390/nu8110741

**Published:** 2016-11-23

**Authors:** Kelei Li, Mark L. Wahlqvist, Duo Li

**Affiliations:** 1 Department of Food Science and Nutrition, Zhejiang University, Hangzhou 310058, China; wfujfqcc@sina.com; 2 Fuli Institute, Zhejiang University, Hangzhou 310058, China; mark.wahlqvist@gmail.com; 3 Monash Asia Institute and Departments of Medicine and of Nutrition and Dietetics, Monash University, Melbourne 3006, Australia

**Keywords:** folate, neural tube defects, vitamin B, choline, betaine, one-carbon metabolism, tea, alcohol, coffee

## Abstract

Neural tube defects (NTDs) are a group of severe congenital malformations, induced by the combined effects of genes and the environment. The most valuable finding so far has been the protective effect of folic acid supplementation against NTDs. However, many women do not take folic acid supplements until they are pregnant, which is too late to prevent NTDs effectively. Long-term intake of folic acid–fortified food is a good choice to solve this problem, and mandatory folic acid fortification should be further promoted, especially in Europe, Asia and Africa. Vitamin B2, vitamin B-6, vitamin B-12, choline, betaine and *n*-3 polyunsaturated fatty acids (PUFAs) can also reduce the NTD risk by interacting with the one-carbon metabolism pathway. This suggest that multivitamin B combined with choline, betaine and *n*-3 PUFAs supplementation may have a better protective effect against NTDs than folic acid alone. Genetic polymorphisms involved in one-carbon metabolism are associated with NTD risk, and gene screening for women of childbearing age prior to pregnancy may help prevent NTDs induced by the risk allele. In addition, the consumption of alcohol, tea and coffee, and low intakes of fruit and vegetable are also associated with the increased risk of NTDs, and should be avoided by women of childbearing age.

## 1. Introduction

Neural tube defects (NTDs) are a group of severe congenital malformations. It is estimated that approximately one out of 1000 newborns present with this type of defect [1]. The development and closure of neural tube happen during normal embryogenesis between the 18th and 28th days after fertilization. Failure of the neural tube to close in embryonic development results in NTDs [2]. The etiology of NTDs is still unknown. One possible reason may be the disturbance of the one-carbon metabolism pathway [3,4]. However, there are also folate-resistant NTDs, indicating that folate deficiency is not the unique reason for NTDs, and other potential pathogeneses may also responsible for NTDs. The deficiency of other nutritional factors involved in one-carbon metabolism, such as vitamin B-2, B-6, B-12, choline, betaine, and *n*-3 polyunsaturated fatty acids, may be also associated with NTDs. Genetic factors are another important cause of NTDs. Many loci on genes have been identified as associated with the risk of NTDs, especially on genes involved in the one-carbon metabolism pathway, such as methylenetetrahydrofolate reductase (*MTHFR*) and 5-methyltetrahydrofolate-homocysteine methyltransferase (*MTR*) [5,6].

In this study, we review the research progress on the effect of nutritional factors and genes involved in the one-carbon metabolism pathway on NTDs. This should provide a basis for better nutritional approaches to NTD prevention.

## 2. B-Vitamins, One-Carbon Metabolism and NTDs

### 2.1. Folate

**Figure 1:**
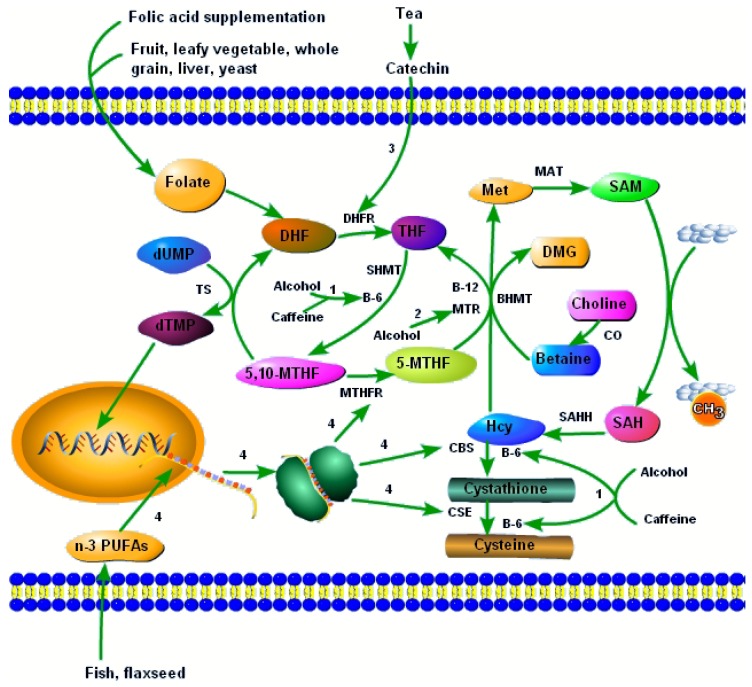
B vitamins and other dietary factors interact with the one-carbon metabolism to influence the development of NTDs. 1, alcohol and caffeine lower vitamin B-6, and thus disturb vitamin B-6–dependent one-carbon metabolism pathways; 2, alcohol reduces the activity of *MTR*, leading to increased Hcy and reduced SAM; 3, catechins in tea reduce the activity of *DHFR*, and hinder the synthesis of THF; 4, *n*-3 PUFAs increase the mRNA expression of enzymes involved in one-carbon metabolism, such as *MTHFR*, *CBS*, and *CSE*. Abbreviations: DHF, dihydrofolate; THF, tetrahydrofolate; 5,10-MTHF, 5,10-methylenetetrahydrofolate; 5-MTHF, 5-methyltetrahydrofolate; dUMP, deoxyuridine monophosphate; dTMP, thymidinemonophosphate; Hcy, homocysteine; Met, methionine; SAM, S-adenosylmethionine; SAH, S-adenosylhomocysteine; DMG, dimethylglycine; *DHFR*, dihydrofolate reductase; *SHMT*, serine hydroxymethyltransferase; *MTHFR*, methylenetetrahydrofolate reductase; *MTR*, 5-methyltetrahydrofolate-homocysteine methyltransferase; *TS*, thymidylate synthase; *BHMT*, betaine-homocysteine methyltransferase; *MAT*, methionine adenosyltransferase; *SAHH*, *S*-adenosylhomocysteine hydrolase; *CBS*, cystathionine-beta-synthase; *CSE*, cystathionine-gamma-lyase.

Folate, also known as vitamin B-9, plays an important role in the homocysteine (Hcy) metabolism, one-carbon pathway and DNA synthesis (Figure 1). In 1991, the Medical Research Council Vitamin Study Research Group conducted a large-scale randomized controlled trial (RCT) with 1817 women from 33 centers in seven countries to assess the effect of folic acid on the reoccurrence of neural tube defects and found that 4 mg folic acid supplementation per day lowered the risk of NTDs by 72% compared with the control group (without folic acid supplementation) [7]. Another important RCT was conducted by Czeizel et al. in Hungarian women without a history of NTD-affected pregnancy [3]. In that study, no NTD cases occurred in 2104 women who received multivitamins containing 0.8 mg folic acid per day, while six NTD cases occurred in 2052 women who received only trace elements and vitamin C. In 1999, Berry et al. conducted a cohort intervention study in an area of China with high rates of neural tube defects (the northern region) and one with low rates (the southern region), and found that preconceptional supplementation (starting supplementation before the last menstrual period before conception and stopping at the end of the first trimester) of 400 μg folic acid (a synthetic form of folate) per day reduced the NTD risk by 85% in the northern subgroup of 31,960 women and by 40% in the southern subgroup of 215,871 women [4]. These results of intervention studies have provided the most persuasive evidence for the protective effect of folic acid against NTDs. 

In 1995, a case-control study by Daly first reported a dose-response relationship between the red blood cell folate concentration of mothers and the risk of NTDs, and that a red blood cell folate concentration ≥906 nmol/L (400 ng/mL) can provide optimal protection against NTDs [8]. In 2014, Crider et al. analyzed data from two intervention studies in Chinese women (400 μg folic acid per day from preconception through the end of the first trimester) by a Bayesian model, and found a dose-response relationship between maternal red blood cell folate and the risk of NTDs: a folate concentration >1000 nmol/L substantially reduced the risk of NTDs [5]. This was consistent with the result of Daly’s study. Based on these results, in 2015 the World Health Organization (WHO) recommended a threshold value of the maternal red blood cell folate concentration of 906 nmol/L (400 ng/mL) to prevent NTDs. Folic acid supplementation is a solution for the insufficient dietary intake of folate. A daily intake of 0.4 mg (at least one month before conception through the first three months of conception) folic acid is recommended by the Centers for Disease Control and Prevention for women who do not have a history of a previous NTD-affected pregnancy; this dose can prevent 50% of NTDs [9]. As for women who have had a previous NTD-affected pregnancy, the dose of folic acid is increased to 4 mg per day (at least one month before pregnancy through the first three months of pregnancy) [9]. Although recommendations for preconceptional folic acid supplementation have existed for decades, only a small number of women were actually supplemented with folic acid before conception [10]. Most women started folic acid supplementation when they knew they were pregnant, and this was often too late for the effective prevention of NTDs [11]. Long-term intake of folic acid–fortified foods should complement preconceptional folic acid supplementation. Arth et al. reported that mandatory folic acid fortification of wheat and maize flour prevented 13.2% folic acid–preventable NTDs (35,500 of approximately 268,700 global cases) in 58 countries [12]. Seventy-eight countries have fortified flour with folic acid mandatorily, while most countries in Asia, Europe and Africa do not mandate folic acid fortification [13]. Khoshnood et al. conducted an observational study to assess the long-term trend in NTD prevalence in 19 European countries and found that, without mandatory folic acid fortification, the long-existent recommendation for preconceptional folic acid supplementation and voluntary folic acid fortification did not significantly decrease the prevalence of NTDs [14]. In 2016, an intervention study in 16,648 women in Shanxi, China (one of the most NTD-affected regions, with a 13.8‰ to 19.9‰ prevalence), found that folic acid–fortified flour decreased the NTD burden by 58.5%, which has informed the future implementation of mandatory folic acid fortification in China [15]. Further promotion of mandatory folic acid fortification is needed to prevent NTDs.

Several genes involved in the folate-dependent one-carbon metabolism have been shown to be associated with the risk of NTDs. One of the most important is the gene encoding *MTHFR*, an enzyme that catalyzes the conversion of 5,10-methylenetetrahydrofolate (5,10-MTHF) to 5-methyltetrahydrofolate (5-MTHF) and provides the methyl group needed for remethylation of Hcy to form methionine (Met) (Figure 1) [16]. The T allele of *MTHFR* 677C>T is associated with an increased risk of NTDs in the western population [17,18,19]. Similar observations are made in Chinese mothers even when they supplement with folic acid [5,16,20]. Two considerations may help explain this association between *MTHFR* 677C>T and NTDs. On the one hand, the TT genotype of *MTHFR* 677C>T can attenuate the plasma and red blood cell (RBC) folate response to folic acid supplementation [21]. In addition, previous studies have reported that the T allele carriers of *MTHFR* 677C>T had lower folate concentrations than non-carriers [18,22]. On the other hand, the *MTHFR* 677C>T mutation is associated with reduced *MTHFR* activity [18,23]. *MTHFR* 1298A>C is yet another mutation associated with decreased *MTHFR* activity [23]. However, the link between *MTHFR* 1298A>C and NTDs remains controversial: one study in Italy reported that the C allele of *MTHFR* 1298A>C was associated with a higher risk of NTDs [24], several other studies found no significant association between *MTHFR* 1298A>C and NTDs [6,25,26,27], while one study in China even found that the C allele of *MTHFR* 1298A>C had a protective role against NTDs [28]. 

In addition to *MTHFR*, other genes involved in the folate metabolism have been demonstrated to influence the development of NTDs in the Chinese. *MTR* is an enzyme that catalyzes the remethylation of Hcy to Met and is dependent on the provision of methyl groups from 5-MTHF (Figure 1). The mutation of *MTR* is associated with the increased risk of NTDs [6,19,29,30]. Solute carrier family 19 member 1 (*SCF19M1*) can transport folate into cells. The mutation of *SCF19M1* is associated with increased NTD risk even when mothers are supplemented with folic acid [6]. In addition, mutations on *BHMT* [6,19,31], *CBS* [19,32], *MTRR* [19,33,34], *MTHFD1* [19,35,36,37,38,39,40,41], *MTHFD2* [19], *SHMT1* [36], *FOLH1* [42], *RFC1* [43,44], *SARDH* [45], *PEMT* [40], *GART* [40] and *TYMS* [19,36] have also been reported to be associated with the risk of NTDs.

### 2.2. Vitamins B-2, B-6 and B-12

Vitamin B-12 is the cofactor of *MTR* (Figure 1). Additionally, B-12 deficiency is associated with elevated Hcy [46,47]. In case-control studies, vitamin B-12 status has been found to be protective against NTDs in the Chinese [47,48]. According to a study in 1170 women in northwest China, the prevalence of vitamin B-12 deficiency was 45% [49], indicating that vitamin B-12 supplementation maybe also be needed to prevent NTDs in China. The negative association between vitamin B-12 and the risk of NTDs is also observed in other populations [50,51,52], and remains significant even in folic acid–fortified populations [53,54]. Transcobalamin II (*TCN2*) is a carrier protein that can bind vitamin B12. The mutation of *TCN2* is associated with an increased NTD risk even when mothers are supplemented with folate [6]. *CUBN* is a gene that encodes the intestinal receptor responsible for the uptake of the vitamin B12–intrinsic factor complex. The mutation of *CUBN* is also associated with the risk of NTDs [40,55]. This genetic evidence also demonstrates the role of vitamin B-12 in the development of NTDs. 

Besides folate and vitamin B-12, vitamin B-2 and B-6 are also important enzyme cofactors involved in the one-carbon metabolism. The conversion of 5,10-MTHF to 5-MTHF is catalyzed by *MTHFR* and depends on FADH2, the hydroquinone form of flavin adenine dinucleotide (FAD) (a derivative of vitamin B-2). Hustad et al., in a cross-sectional study, found that plasma vitamin B-2 was negatively associated with Hcy [56]. An intervention study by McNulty et al. in healthy adults found that vitamin B-2 supplementation lowered the concentration of Hcy only in subjects with a TT genotype of *MTHFR* 677C>T, but there was no effect in CC or CT genotypes [57]. In addition, vitamin B-2 can interact with folate to modulate Hcy concentrations. One intervention study found that folic acid supplementation (400 μg/day) had a greater Hcy-lowering effect in subjects with a high plasma level of vitamin B-2, and this effect was unrelated to *MTHFR* 677C>T polymorphism [58]. Several intervention studies have found that preconceptional multivitamin supplementation (including vitamin B-2 and several other vitamins such as folic acid, vitamin B-6, vitamin B-12, vitamin E, thiamin, vitamin A, vitamin D, nicotinamide, and ascorbic acid) could reduce the risk of NTDs [3,59]. Vitamin B-6 is the cofactor for betaine-Hcy methyltransferase (*BHMT*) (an enzyme that catalyzes the remethylation of Hcy to Met with betaine providing the methyl needed), cystathionine-beta-synthase (*CBS*) (an enzyme that catalyzes the reaction from Hcy to cystathionine) and cystathionine-gamma-lyase (*CSE*) (an enzyme that catalyzes the reaction from cystathionine to cysteine) (Figure 1). Vitamin B-6 deficiency is also associated with increased Hcy [46]. A French case-control study found that maternal plasma vitamin B-6 was negatively associated with NTD risk [33]. Thus, supplementation of vitamin B-2, vitamin B-6 and vitamin B-12 together with folic acid may have a better protective effect against NTDs than folic acid supplementation alone. However, this still needs to be demonstrated by well-designed intervention studies.

### 2.3. Potential Adverse Effects of B Vitamins

Despite the beneficial effect for NTD prevention, observational studies showed that very high doses of folic acid supplementation during conception also have several adverse effects. However, the evidence is still inconsistent. Observational studies showed that folic acid supplementation during conception was associated with the increased risk of wheezing in children through 18 months of age (dose was not reported) [60] and the increased risk of infant asthma (>72,000 μg∙day) [61]. Evidence from observational studies showed that folic acid supplementation was associated with the increased risk of infant clefts (dose was not reported) [62] and spontaneous preterm delivery (mean dose (interquartile range): 313 (167–558) μg/day) [63]. Valera-Gran et al. found that high folic acid supplementation for mothers during conception (>5000 μg/day) had a detrimental effect on the psychomotor development of children [64]. However, Tolarova et al. found that preconceptional supplementation of 10 mg/day folic acid plus multivitamins significantly reduced the risk of infant clefts [65]. One population-based cohort study in China found that preconceptional folic acid supplementation (400 μg/day) significantly reduced the risk of spontaneous preterm delivery [66]. McGarel et al. reported that folic acid supplementation had a beneficial effect on brain development and cognitive performance [67]. The FDA’s safe upper limit of folic acid is 1000 μg [68]. Folic acid is the synthetic form of folate. Before entry into the circulation system, folic acid undergoes reduction (by DHFR) and methylation to 5-MTHF, the circulating form of folic acid. However, when folic acid supplementation exceeds a certain dose, other transport mechanisms, such as passive diffusion, will complement the normal absorption mechanism, and thus unaltered folic acid enters the circulation system [69]. That is unmetabolized folic acid. One acute study found that folic acid supplementation of more than 800 μg/day can cause unmetabolized folic acid accumulation in the serum, but when the dose was no more than 400 μg/day, the unmetabolized folic acid in the serum was undetectable [69]. A prospective study of pregnant Canadian women found that unmetabolized folic acid was detectable in more than 90% of maternal and cord plasma samples, which may be a result of excess folic acid supplementation [70]. One RCT found that folic acid supplementation with a dose of 400 μg/day during conception had no significant influence on the unmetabolized folic acid concentration in maternal plasma or newborn cord blood plasma [71]. Therefore, a dose of 400 μg/day is safer than 1000 μg/day for folic acid supplementation. 

Bailey reported that a higher vitamin B-6 intake (>1.85 mg/day) of mothers during the last six months of pregnancy was associated with a higher risk of childhood lymphoblastic leukemia [72]. However, one case-control study found that the per 1 mg increase of preconceptional vitamin B-6 intake from food and supplements was associated with a 11% decreased risk of childhood acute lymphoblastic leukemia. As suggested by US authorities, the level of vitamin B-6 with no observed adverse effect is set at 200 mg per day while the safe upper limit is at 100 mg per day [73]. Supplementation of vitamins B-2 and B-12 has not been associated with any adverse effects. Additionally, there is still insufficient evidence to set safe upper intake levels for vitamin B-2 and vitamin B-12 [74].

## 3. Choline, Betaine, One-Carbon Metabolism and NTDs

Choline is a nutrient associated with NTDs. Food sources of choline are principally those rich in lecithin (phosphatidylcholine) such as eggs and soy beans [75]. Betaine, which may be derived from choline, is found mostly in green leafy vegetables and beets (root vegetables). Choline can be synthesized in vivo by the methylation of phosphatidylethanolamine (PE) to phosphatidylcholine (PC) [76]. However, its biosynthesis is limited and dietary intake of choline is necessary [76]. Choline can be metabolized to betaine, catalyzed by choline oxidase (Figure 1). Similar to 5-MTHF, betaine can also provide the methyl needed in the remethylation of Hcy to Met, a reaction catalyzed by *BHMT* (Figure 1). The mutation of *BHMT* has been demonstrated to be associated with the risk of NTDs [6,19,31]. The ability of betaine supplementation to lower Hcy has been reported in the Netherlands [77]. Abnormal choline metabolism can lead to NTDs in the mouse [78]. An observational study found that preconceptional dietary intake of choline and betaine was negatively associated with the risk of fetal NTDs independent of folate intake in Americans [79]. 

However, choline and betaine intake can adversely affect serum lipid concentrations, such as increase total serum cholesterol, low density lipoprotein (LDL), high density lipoprotein (HDL) and triacylglycerol. Through the gut microbiome and trimethylamine production, they can also increase the risk of atherosclerotic vascular disease [80]. The safe upper level of choline may be 3500 mg/day for people with an age ≥19 years [81]. The safe upper level of betaine is unknown.

## 4. Other Dietary Factors Interact with the One-Carbon Metabolism to Influence the Development of NTDs 

A case-control study has found that preconceptional tea consumption increases the risk of NTDs in Shanxi, China [82]. Similar findings have been observed in a case-control study in Atlanta [83]. In another case-control study conducted in the US, tea consumption was not associated with the risk of NTDs; however, when subjects were divided into subgroups according to the dose of folic acid intake, tea consumption was associated with an increased risk of NTDs in subjects with a folic acid intake >400 μg, and the authors suggested that tea consumption might interact with the folate metabolism pathway to influence the occurrence of NTDs [84]. The effects of tea consumption on the blood folate level are controversial [85,86]. One study has reported a lowering effect of tea consumption on blood folate [86]. A related case-control study found an association between the polymorphisms of catechol-*O*-methyltransferase (*COMT*) (encoding the enzyme that catalyzes the methylation of catecholamines with S-adenosylmethionine (SAM), a methyl donor) and the risk of NTDs in the Chinese: the mutant homozygotes of rs73,785 or rs4633 had a higher risk of NTDs than the wild homozygotes did, while the heterozygotes of rs4680 had a lower risk of NTDs than the wild homozygotes did, and the rs4680 genotype interacted with tea drinking to alter the risk of NTDs [87]. The effect of tea drinking on the development of NTDs can be explained by an inhibitory effect of tea catechins on dihydrofolatereductase [88]. Dihydrofolatereductase (*DHFR*) is an enzyme that catalyzes the conversion of dihydrofolate (DHF) to tetrahydrofolate (THF), the active form of folate. THF plays an important role in homocysteine metabolism and thymidine monophosphate (dTMP) synthesis. In summary, tea catechins inhibit the activity of *DHFR*, thus blocking DNA synthesis and homocysteine clearance, and this may help explain the association between tea consumption and the risk of NTDs (Figure 1). 

One case-control study in Italians found that alcohol, low fruit and vegetable intake, and coffee were associated with an increased risk of NTDs [89]. Alcohol consumption is associated with lower blood folate as well as pyridoxal 5′-phosphate (the active form of vitamin B-6) and higher Hcy [90]. Animal studies showed that alcohol consumption can reduce the level of SAM (the major methyl donor) [91,92] and the activity of *MTR* (the enzyme catalyzing the remethylation of Hcy to Met) (Figure 1) [93]. Fruit and vegetables are rich in folate, and thus a low intake of these foods may be associated with inadequate folate intake (Figure 1). In pregnant Japanese women, it has been found that caffeine intake is associated with elevated Hcy only in subjects with a high intake of vitamin B-6 [94]. The positive association of caffeine with the level of Hcy and the risk of NTDs can be attributed to it having a similar chemical structure to theophylline, which can decrease pyridoxal 5′-phosphate (the active form of vitamin B-6) by acting as an inhibitor of pyridoxal kinase (Figure 1) [95,96]. 

In addition, *n*-3 PUFAs can lower Hcy [97,98,99,100], and upregulate the expression of several enzymes involved in the one-carbon metabolism, such as *MTHFR*, *CBS*, and *CSE* (Figure 1) [101]. Our unpublished case-control study in the Chinese has found that placental C18:3*n*-3, C20:5*n*-3 and C22:5*n*-3 are negatively associated with fetal NTD occurrence. The meta-analysis indicates that *n*-3 PUFAs combined with vitamin B supplementation have a greater Hcy-lowering effect than *n*-3 PUFAs alone [100]. However, there has been a concern that high-dose *n*-3 PUFA supplementation might induce bleeding [102]. However, there is little evidence to support this [103,104,105]. Previous studies showed that marine-derived *n*-3 PUFAs supplementation can promote LDL oxidation [106], but this is still controversial [107].

## 5. Perspective and Prospects 

Although preconceptional folic acid supplementation has been recommended for decades, its overall ability to reduce the prevalence of NTDs is limited. Many women do not take a folic acid supplement until they are pregnant, which is too late for the effective prevention of NTDs. Long-term intake of folic acid–fortified foods should complement preconceptional folic acid supplementation. However, mandatory folic acid fortification is still not universally in place in Europe, Asia or Africa. However, folate-resistant NTDs exist, so there are other reasons for NTDs other than folate deficiency. In recent years, studies have indicated that the low intake and status of vitamin B-2, B-6, B-12, choline, betaine or *n*-3 PUFAs, and the consumption of alcohol, tea, or coffee are also associated with an increased risk of NTDs. Further cohort and intervention studies are needed to demonstrate whether multivitamin B (folate, vitamin B-2, B-6, B-12) combined with choline, betaine and *n*-3 PUFAs, or simply a biodiverse diet, has a better protective effect against NTDs than folic acid alone. In addition, genetic factors play an important role in the development of NTDs. There are many genetic variants involved in the one-carbon metabolism demonstrated to be associated with the risk of NTDs. Some variants can increase the risk of NTDs regardless of folic acid supplementation. Therefore, gene screening of women of childbearing age prior to pregnancy could enhance efforts to prevent NTDs.

## 6. Conclusions 

Further cohort and intervention studies are needed to demonstrate whether multivitamin B (folate, vitamin B-2, B-6, B-12) combined with choline, betaine and *n*-3 PUFAs supplementation, or a biodiverse diet, has a better protective effect against NTDs than folic acid alone. Mandatory folic acid fortification and nutrition education, targeted at women in the reproductive age group, should be promoted and gene screening for women of childbearing age prior to pregnancy should be made available to prevent NTDs. These strategies would help decrease the burden of this oppressive health problem, especially in high-risk populations.
